# Notes and Comments

**Published:** 1894-02

**Authors:** 


### Notes and Comments.[1]

PULP-CAPPING.—Dr. Charles Harker has no confidence in pulp-capping when the exposure results from caries, but, on the contrary, says pulps will frequently die when capped under the most favorable circumstances; and he is further of the opinion that "a tooth-pulp, instead of being easily manageable, is so treacherous that we can never predict with certainty the result of capping."

The writer cannot entirely agree with Dr. Harker in what he says upon this subject. While it is true that we can seldom predict "with certainty" the result of capping, yet so large a percentage of cases in an extensive clinical experience have given such satisfactory results that we always consider it the best practice in favorable cases to cap the pulp, and at least give it an opportunity to live.

---

MIXING AMALGAM.—Dr. Mayer, in his paper before the New York Odontological Society, upon "The Working Qualities of Amalgams," says he does not believe in working the amalgam as dry as some advocate. He wants it plastic enough to pack well, and likes to have it packed before crystallization has far advanced.

This has always been our practice. We never advocate the system of using pliers to force out the mercury so as to leave it in

---

[1] The assistant editor solicits contributions for this department,—new methods, new remedies and formulas, or any short practical note which may prove of value to the practitioner or student. Address 1506 Arch Street, Philadelphia.

a dry and almost unmanageable condition. By working it in a moderately plastic state, all surplus mercury will be forced to the surface in packing the filling, which can be removed from time to time with a small pledget of cotton.

---

Dental Service to the Insane.—A timely editorial appears in a recent issue of the *Southern Dental Journal* upon the appointment of dental surgeons in State lunatic asylums. This matter should not be allowed to stop here. When we consider the intimate relationship and sympathy existing between the teeth and the nervous system, it seems strange that some action has not long ago been taken for the relief of this unfortunate portion of our people.

Professor Wilbur F. Litch, in writing upon the subject, says, "I have repeatedly operated for private patients in the insane department of the Pennsylvania Hospital, and there can be no question as to the necessity for the services of a skilful dentist in all such institutions. Mental diseases cannot fail to be aggravated by pathological conditions of the teeth, and the trained dentist alone is qualified to diagnosticate and treat such ailments."

---

The Treatment of "Quacks."—We append the following resolution as a suggestion to our professional schools. If some such action and public announcement of the same should be taken by the Faculties of dental colleges where one whom they had honored with a diploma brings discredit upon the profession and their Alma Mater, it would in our opinion have a wholesome effect upon the profession and the public.

The following preamble and resolution were adopted by the Faculty of the medical department of the University of Tennessee, Nashville Medical College, at a meeting held at the office of Drs. Duncan and Paul F. Eve, September 2, 1893:

Whereas, It appears that one E. F. Rose, of Dukedom, Tennessee, having complied with the requirements for graduation, received the degree of M.D. from the Medical Department, University of Tennessee, Nashville Medical College, in 1892; and

Whereas, It has been made known and positively proved to the satisfaction of this Faculty that the said Dr. Rose has so far forgotten and ignored the principles of honorable medicine as to enter into all manner of advertising schemes to obtain practice, in violation of the code and professional decency, etc.; therefore be it

*Resolved*, That we, the Faculty of the Medical Department, University of

Tennessee, Nashville Medical College, censure and condemn the conduct of the said E. F. Rose as unprofessional and injurious to the college from which he graduated to practise medicine, and that the dean be instructed to strike his name from the records as an alumnus, and declare him beyond the role of professional recognition.

---

"To do more and be more, or to do less and be less, is compulsory; a forward movement or retrogression is inevitable. All things retrograde which do not advance, is an axiom which applies alike to the college, the periodical, the society, and to the individual."

---

Professional Fees.—To serve our clients best we must show a personal interest in each individual case, and remember that we are laboring not for the fee alone, but for the love and advancement of our profession. Remember the well-chosen words of Edward Everett Hale, where he says, "Professional men must serve the world not, like the handcraftsman, for a price accurately representing the work done, but as those who deal with infinite values and confer benefits as freely and nobly as nature."

---

The Successful Dentist.—"The nimbleness and dexterity of a skilful dentist is wonderful. The very instruments and gold he uses seem alive. As he stands behind them they obey his will so promptly and with such precision they seem a part of himself. Young man, would you attain such an enviable position? Work for it with brain and muscle; sacrifice for it every foolish habit, every nonsensical indulgence, every hindering pleasure; give your life for it; bury yourself in its necessities till you can come forth transformed by the regenerating power of study and delicate manipulation."—*Items of Interest.*

---

Some of the Necessities for a Creditable Literature.—The International Dental Journal is not published to advance the business interests of the publishers nor the professional success of its editors. They are all free from any such collateral necessity. But some one must do the work; some one must make sacrifices if we are to have a journal freely our own. Every one who is interested in the advance and elevation of dentistry should show a personal and professional interest in the welfare of its representative.

The best way to sustain a creditable literature is for every intelligent dentist to contribute what he can. Every practitioner worthy the name may have some thought or experience stored up that would be helpful to others. There are a few gifted men who contribute volumes to our literature, but there are hundreds of us who could enrich it with a page or a paragraph. This department is always open for such contributions. All are welcome, but there is one suggestion that we would offer: that is, in recording your observations let it always be with accuracy and truthfulness. Do not set down your own theories and speculations as absolute facts and take for granted things that are undemonstrable.

